# Graduate Global Public Health Education: Activities and Outcomes in Relation to Student Prior Experience

**DOI:** 10.5539/gjhs.v5n3p54

**Published:** 2013-01-31

**Authors:** Suzanne F. Jackson, Donald C. Cole

**Affiliations:** 1 University of Toronto, Toronto, Canada

**Keywords:** public health training, graduate education, global health, competencies

## Abstract

The Dalla Lana School of Public Health uses an “add-on” or concentration model of global health education. Records of masters’ graduate cohorts across five disciplinary fields from 2006 to 2009 were classified as to prior experience at application and completion of global health concentration requirements. Alumni from the first two cohorts (2006-08 and 2007-09) were interviewed using a semi-structured interview guide. Prior experience was not linked consistently with the number of elective courses, location of practica or completion of requirements. Successful completion of the global health requirements depended more on the student’s base disciplinary program. Interviewed alumni with medium prior experience reported greater satisfaction with the concentration. Alumni with lower prior experience wanted more courses and support with practica. The pros and cons of a concentration model of global public health graduate education are discussed.

## 1. Introduction

The world has become an interdependent global community, sharing similar health issues and public health functions. A global approach is needed to face the issues and carry out actions (Conrad, Mazet, Clifford, Scott, & Wilkes, 2009; Frieden & Henning, 2009). “Global health is an area for study, research, and practice that places a priority on improving health and achieving equity in health for all people worldwide. It emphasizes transnational health issues, determinants, and solutions; involves many disciplines within and beyond the health sciences and promotes interdisciplinary collaboration; and is a synthesis of population based prevention with individual-level clinical care” (Koplan et al., 2008).

In many leading educational institutions in high income countries, global health programs are flourishing. Institutions have provided not only on campus programs but internet-based courses offering useful and timely continuing education for health professionals off campus (GHeL-USAID). Over the last decade, public health educators at universities have faced increasing interest in global public health among incoming graduate students, perhaps best documented in US Schools of Public Health (SPH) (Hagopian et al., 2008). Involvement in international health, or more recently global health has been a feature of public health professional activity (Koplan et al., 2009).

Much has been written about incorporating global health into medical education (Crump & Sugarman, 2008; Vermund, Audet, Martin, & Heimburger, 2010; Drain et al., 2007; Godkin & Savageau, 2003; Stapleton, Wahl, Norris, & Ramsey, 2006) but there is little written about the education of public health practitioners at the graduate level (Sadana, Mushtaque, Chowdhury, & Petrakova, 2007; Frenk et al., 2010). Public health is emerging as an important field globally given pandemics, maternal and child health issues, and the presence of concurrent infectious and chronic diseases. These all require public health interventions and an understanding of globalization trends and interactions. Health promotion, epidemiology, nutrition and biostatistics are necessary skills for addressing global health issues. Thus it is important for non-clinicians who want to work globally to get training in basic useful skills as well as training in the special aspects of working in a globalized world.

In Canada, at least 10 university public health schools or departments (SPH) are offering graduate education for public health students interested in global health, using different models (see Box 1). Given the limited literature on global education at the graduate level (Stearns, 2009), and the investments involved in developing such programs, it is important to understand the strengths and weaknesses of these different models. The dedicated Masters programs in global health have the advantage of creating in-depth knowledge of globalization processes and providing specific global health practicum experiences. The drawback is that the graduates are perceived to have more generalist skills when organizations are looking for specific public health expertise. In the “support” model, students are supported in finding placements overseas and in applying concepts to global health problems in their courses but there are no consistent program requirements. The add-on or collaborative concentration in global health has the advantage of building a generic disciplinary skill set (most commonly epidemiology) that can be applied in a variety of settings, including global ones. The drawback is that the student has enough space in their program for only a few global health courses, leading to a lack of in-depth knowledge in global health.

**Box 1:** Canadian university graduate offerings in global health as of mid-2010

***Support programs*** for graduate students, medical students and faculty doing work in global health –
McGill (http://www.mcgill.ca/globalhealth/)
Dalhousie University (http://gho.medicine.dal.ca/about/index.htm)
University of Manitoba (http://umanitoba.ca/faculties/medicine/units/community_health_sciences/departmental_units/cgph/about.html)
Western Ontario (http://www.schulich.uwo.ca/globalhealth/index.php)
Saskatchewan (http://www.medicine.usask.ca/dean/cc_social_acc/international-programs)

***Add-On or Collaborative concentration*** in global health –
Simon Fraser University (http://www.fhs.sfu.ca/graduate-programs/master-of-public-health-mph-program/global-health-concentration)
University of Toronto (http://www.phs.utoronto.ca/masters_global_health.asp),

***Dedicated master’s programs*** that grant MPH or MSc degrees in global health –
University of Alberta (http://www.phs.utoronto.ca/masters_global_health.asp),
Université de Laval (https://capsuleweb.ulaval.ca/pls/etprod7/y_bwckprog.p_afficher_fiche?p_session=201009&p_code_prog=M-SAC&p_code_majr=SML&p_code_camp=&p_type_index=4&p_valeur_index=0)
McMaster University (http://www-fhs.mcmaster.ca/global_health_masters/msc_degree_requirements.html).

Our hypothesis is that each model suits a different kind of student. A student with little prior experience in global health may benefit from a dedicated program focused on teaching a set of skills and providing experiences that will support the student in beginning a career in global health. The other two models may be better suited for students who already have some experience and need specific public health disciplinary skills in order to further their existing career in global health.

In this paper, we focus on the add-on or concentration model at the University of Toronto, asking the question “Are certain students more suited to the GH concentration model of education?” We report assessment of prior experience in global health at the time of application and we examine completion of global health concentration requirements (derived through review of program monitoring files) by prior experience, as well as course and practica in one field (Health Promotion). In addition, the interviews of the first two alumni cohorts focus on their assessments of the impact of the concentration on their learning, careers, and perceived competencies in global health, along with suggestions for improvement. At the end, we reflect upon the implications for graduate global public health education.

## 2. Area Description: The Global Health Concentration Model at University of Toronto

The Global Health (GH) concentration at the University of Toronto’s Dalla Lana School of Public Health (DLSPH) is added into disciplinary based Masters of Public Health (MPH) programs of 20 months duration with part-time and full-time study options. When the concentration was originally conceived in 2004-5, there were debates among faculty about which model to adopt. The three options discussed were whether to create a special field or stream focused on global health (dedicated program model), create a collaborative program enabling students in all public health disciplines to gain from exposure to consistent course requirements and an identified cohort of students, or building global health into all courses in all disciplines so that the entire focus of the DLSPH would be on global health as well as each discipline. Given the lack of global health expertise in all faculty, and a strong argument that our strength was our disciplinary training, the collaborative concentration model was selected. Global health training was designed as a secondary emphasis built into the elective space of students’ primary public health program.

MPH applicants accepted into primary disciplinary MPH programs underwent a second selection based on an assessment of prior international health related academic studies, experience (volunteering or work in international or disadvantaged settings) and interests (global health as a major part of career aspirations).

The GH concentration’s three requirements have been completion of:

a) the core course in Global Public Health (GPH) taken as an elective;
b) one elective course in global/international health from anywhere in the university; and
c) one practicum placement with a global health focus.

Only the first requirement was restricted to GH concentration students, with the result that students who were not accepted or dropped the concentration could still do (b) and (c). Monthly GH concentration meetings were a non-required opportunity, at which attendance varied. The details of recommended electives, timing and the practicum options were tailored to the needs of each discipline (including certification requirements for some fields e.g. community nutrition) in consultation with the respective base Program Directors. For example, the duration of the practicum placement differed in length and focus for each disciplinary base (or primary) program.

## 3. Methods

All student applications to the global health concentration from 2006 to 2009 were assessed on prior experience using criteria for length of time volunteering or working in non-Canadian settings, number of international health courses taken as an undergraduate, and interest/understanding of the social determinants of health. Although categorization differed slightly from year to year, admission reviewer ratings of high, medium high, medium, low and very low prior experience levels could be compared across admission years (Table 1). The program monitoring files were also reviewed to extract the number of international health course electives completed and the location of practica for each student, and whether students completed the core course in global public health. The completion of these requirements was examined in relation to prior experience categories and associations with prior experience examined using Fisher’s Exact Test.

**Table 1:** Prior experience categorization, criteria and numbers of students at admission

Category of Prior Experience	Criteria	Number of Students (Admission Years 2006-2009)
High	Worked or volunteered in another country for more than 6 months + had degree in international development + understands social determinants of health well	22 (including 2 withdrawals)
Medium High	Worked or volunteered in another country for 3 weeks to 6 months + >2 courses in international health + can describe factors beyond treatment that affect health	13 (including 1 withdrawal)
Medium	Worked or volunteered in another country for 3 weeks to 6 months + 1-2 courses in international health	19 (including 2 in process)
Low	Travelled to other countries and worked for an International NGO in Canada + 1 undergrad course in international health	8
Very Low	No experience overseas, no international health courses	5 (including 2 in process)
Data missing so unable to classify prior experience		16 (including 3 withdrawals and 1 in process)
TOTAL		83

(Data for table drawn from admission reviewer ratings – required to determine admissibility into Global Health Concentration)

In addition, we invited all alumni from the first two GH concentration admissions (2006 and 2007), those who applied to the GH concentration but were not admitted and those who completed requirements or who dropped the GH concentration to be interviewed. Initial contact was by email through a letter from faculty members most involved in the concentration. Ethics approval was received from the Ethics Review Board at the University of Toronto. A Research Assistant contacted alumni by email, telephone, or Facebook up to three times to arrange an interview.

The semi-structured interview guide aimed to tap experience before entering the masters, aspects of the masters’ experience including each curriculum component and activities since graduation. Telephone interviews with alumni were tape-recorded with permission, consent to participate was obtained before the tape was turned on, the interviews were all conducted in English, and the qualitative sections were transcribed. De-identified qualitative data were reviewed by the team for key themes and then individual interviews were coded by the Research Assistant (thematic analysis by question). Given that the faculty members were still providing references for alumni and that individuals could be identified by their responses to certain questions, the respondents asked that the qualitative answers be de-linked. Thus, the Research Assistant did all primary coding, and prepared summary files of the qualitative excerpts without identifiers in each thematic category so they could not be linked to the other responses. Numerical data included alumni self-ratings of GH competencies using a 0-10 Likert scale. Quantitative cross-tabulations were prepared by the Research Assistant including prior experiences, GH program components, learning and career outcomes. Faculty reviewed the cross-tabulations in Excel looking for patterns. The qualitative answers to the question about alumni opinions about the Concentration Model were thematically analyzed and described in depth in this paper.

Response to the Alumni Survey (Table 2) was acceptable overall (63%) with the highest proportions among alumni from the fields most involved in the GH concentration, Health Promotion (71%) and Epidemiology (100%). Perhaps understandably, response was lowest among those who had dropped the concentration (33%).

**Table 2:** Alumni Interviewed from 2006 and 2007 admissions by field and status

Status	No. Alumni	No. Interviewed (response proportion)
In GH Concentration by field		
Health Promotion	14	10 (71%)
Epidemiology	6	6 (100%)
Other (Community Nutrition, Occupational Environmental Health, Family and Community Medicine)	5	2 (40%)
Not in GH Concentration		
Applied but Not accepted	10	6 (60%)
Dropped	6	2 (33%)
Totals	41	26 (63%)

## 4. Results

### 4.1 Prior International Experience

Of the students in all admission years, most students had a high or medium level of prior experience (volunteer or work experience overseas and undergraduate coursework) (see Table 1). Some students in 2006 and 2007 not in the GH Concentration went on global health practicum placements with mixed success. It was felt that admitting all students who wanted to be in the concentration would be beneficial over the long term. Admission of students in low and very low categories thus occurred in 2008 and 2009 because the selection cut-offs were loosened up.

### 4.2 Completion of Global Health Concentration Requirements

According to the review of program monitoring files, the proportions of those graduating with the global health concentration were very good for the first two years: 85% of the 2006 cohort, 80% of the 2007 cohort – though lower in subsequent years - 70% of the 2008 cohort, and 41% of the 2009 cohort. Of the 31 students who did not meet the global health concentration requirements between 2006 and 2009, the most frequent reason was that they did not take the core course or complete even one global health elective.

Even though 2009 had the greatest number of students who were categorized as low or very low at admission, this was not the group that contributed the most to the 59% non-completion rate for that admission year. The greatest numbers who did not meet the program requirements from the 2009 admission year were in high and medium prior experience categories.

The majority of health promotion and 100% of community nutrition and family and community medicine students completed the GH concentration compared to approximately one-third of epidemiology and occupational/environmental (OEH) students. Of those in epidemiology who did not complete, most of them had high or medium prior experience. There are no clear patterns and the reasons for not completing seem to have more to do with the desire of epidemiology and OEH students to meet the base program requirements and increase their specialized skills. Anecdotally, several students with medium to high prior experience stated their desire to increase their public health skills. They felt that they had enough prior experience to move them forward to a career in global health without a GH concentration as part of their degree. In Table 3, even though the proportion of those dropping the concentration is very high in all other streams compared to that of the health promotion students (who were the largest single cohort - 39 for health promotion versus 33 for all other streams), the connection to prior experience ratings was not significant.

**Table 3:** Completion of global health requirements by program stream and prior experience (Admission years 2006-2009, N = 83)

Prior Experience	Health Promotion		All Other Streams*		Totals

	Complete	Didn’t Complete	Complete	Didn’t Complete	
High	11	2	3	4	20
Medium/Medium-High	15	2	5	7	29
Low/Very-Low	5	1	3	2	11
Missing	3	0	7	2	12
Totals	34	5	18	15	72

* All Other Streams = Epidemiology, Community Nutrition, Occupational and Environmental Health, Family and Community Medicine.

Prior Experience data drawn from admission reviewer ratings and completion assessment based on program monitoring files kept for each student. Other students: in progress = 5, withdrawn = 6 Overall non-significant: Fischer's Exact test for count data, p-value = 0.4181

Reasons for not meeting GH requirements cited in the alumni survey were that their primary public health program made heavy demands on their time, they wanted to focus more on experiences and electives related to their base public health program, they did not complete the MPH, or they were dissatisfied with the GH concentration. In the alumni follow-up, those with high international experience disproportionately dropped the GH concentration (Table 4, right hand column) and reported dissatisfaction on interview.

**Table 4:** Prior experience of interviewed alumni (2006 & 07 admission years) in and outside GH concentration

Prior	In GH Concentration (N=18)	Not accepted into or dropped GH Concentration (N=8)
**Training**	4 MDs	2 clinicians
	2 international development	3 international development
	6 biological/health/nutritional sciences	1 neurosciences
	5 social sciences	2 social sciences
	1 other	

**GH Prior Experience**		
*High or medium high*	3	2 dropped and 1 not accepted
*Medium*	5	0
*Low*	6	2 not accepted
*Very low*	4	3 not accepted

Data drawn from Admission Reviewer Ratings and notes made on each student as part of the admission review

### 4.3 Prior Experience, Courses, Competencies, and Careers

Given the association between degree of prior experience in global health with dissatisfaction expressed by those in the alumni follow-up study, we hypothesized that there would be other associations based on level of prior experience. Since we had the most complete data on file for health promotion (HP) students from 2006 to 2009, we examined their selection of international electives and practicum placements. HP students have room in their curriculum to select five course electives after meeting the core requirements for health promotion and the core course for global health. Higher proportions of HP students with medium to high prior experience (38% to 55%) from 2006 to 2009 selected two international or global health course electives and 38% of those with high prior experience were satisfied with one elective (Table 5). Given that one-third of the HP students had high prior experience, this information suggests that the students may be focusing their attention on building other skills because they already have a lot of prior GH experience. On the other hand, the proportion of HP students taking three or more international health courses (25% to 33%) was higher in those with low to medium-high prior experience. It makes sense that students with lower experience would want to take more courses but the pattern is not definitive.

**Table 5:** Global Health Course Electives Completed among Health Promotion Students by Prior Experience (Admission years 2006-2009, N=36)

Prior Experience	One	Two	Three or More	Total
High	5	6	2	13
Medium-High/Medium	4	8	5	17
Low/Very-Low	3	1	2	6
Total	12	15	9	36

Prior Experience data drawn from admission reviewer ratings and number of course electives drawn from program monitoring files kept for each health promotion student Overall non significant: Fischer's Exact test for count data, p-value = 0.5739

Health promotion students have one required 12-week practicum and one optional 16 week practicum placement, either or both of which could be a global health placement for those in the GH concentration. A placement in Canada could be considered a global health placement if it was linked to work with Aboriginal or immigrant and refugee populations, it involved analysis of an international dataset, or the work was for an organization in Canada conducting work internationally (e.g. MSF, World Vision). Given the lack of funding for overseas placements, many students opted for such local GH placements. Close to one-third of HP students (N=35) did both placements designated as global health, 57% did one placement, and 11% did no GH placement (Table 6). Even though more than half the HP students with high, medium high and low prior experience did only one GH placement and equal numbers of those with medium prior experience did one or two GH placements, the role of prior experience in making these choices was not statistically significant.

**Table 6:** Global Health-Related Practicum Experiences for Health Promotion Students by Prior Experience (Admission years 2006-2009, N=36)

Prior Experience	Number of Global Health Practica (Overseas or Local Global)			Total

	Zero	One	Two	
High	1	8	4	13
Medium-High/Medium	2	9	6	17
Low/Very-Low	1	4	1	6
Total	4	21	11	36

Prior Experience data drawn from admission reviewer ratings and type of practicum based on program monitoring files kept for each health promotion student Overall non significant: Fischer's Exact test for count data, p-value = 0.9585

Again, one can hypothesize that those with more prior experience had less need to do both placements overseas, whereas those with low experience could be using the placements to gain experience in global health. Anecdotally, many students chose placements in Canada for different reasons: (a) need to take courses at same time as practica; (b) need to be supervised by Canadians to meet the certification requirements of their program e.g. community nutrition; and (c) lack of ongoing funded placement opportunities internationally. We observed that some students with prior experience overseas used their connections to find practicum placements for themselves or their student colleagues.

From the interviews, alumni from 2006 and 2007 admission years with medium experience reported getting the most from the required GPH course. The global health elective courses were not rated highly and did not contribute to learning for 50-60% of alumni (in GH concentration or not). International placements were more common among alumni with medium experience or higher and those alumni reported that such placements contributed to their learning.

Faculty identified six major areas of competence which we felt were required of public health workers from any discipline when working globally (Table 7, left hand column). When asked to what extent the GH concentration had adequately addressed these six GH competencies (1 low to 5 high), alumni from the 2006 and 2007 cohorts most commonly rated the neutral category (3/5). For their self-ratings of competencies (0 low to 10 high), “be sensitive to cultural differences and adapt methods to local contexts” was highest among the alumni whether in the GH concentration or not, (right hand column, Table 7). On their ability to “understand the political economy of global health issues,” GH concentration alumni rated themselves higher than those not accepted or who dropped out (though with wide standard deviations), perhaps because the Global Public Health core course emphasized the political economy of health.

**Table 7:** Alumni self-ratings (0 low to 10 high) on global health masters’ concentration competencies (n=26)

Competencies– to be able to:	GH Concentration Alumni (mean, SD)	Non-GH concentration Alumni (mean, SD)
1. Understand the political economy of global health issues.	6.9 (1.9)	5.6 (2.7)
2. Bring a determinants-of-health and population health perspective to problem analysis, policy development and project design.	7.9 (1.8)	7.6 (1.1)
3. Be cognizant of the linkages between local & global health problems.	7.9 (1.3)	7.8 (1.5)
4. Work within the mandates, roles and approaches of international organizations.	7.2 (2.0)	7.7 (1.7)
5. Build coalitions and work in partnership with the NGO sector and local community organizations.	7.5 (1.8)	6.7 (2.2)
6. Be sensitive to cultural differences and adapt methods to local contexts.	8.3 (1.1)	8.2 (1.4)
7. Understand broad ethical issues as they relate to equity globally.	8.1 (0.9)	7.8 (1.0)
8. Apply appropriate ethical approaches to international, country level and local projects.	7.4 (1.6)	6.8 (1.7)

Overall, 17% of GH respondents said that the concentration expanded their perspective or changed the lens through which they saw GH problems. Unfortunately, more (28%) felt that the global health concentration did not contribute any additional relevant skills (with no pattern linked to level of prior experience), perhaps due to the restricted opportunities for global health specific activities via placements or coursework, given the heavy requirements of their base programs. Some GH concentration alumni stated that they deliberately changed their mind to focus on a career in Canada. The majority of alumni, whether in the GH concentration or not, stated that their primary disciplinary program connected them to their careers.

### 4.4 Alumni Views of the Concentration Model

Among the alumni interviewees, three major issue areas were identified about the concentration model – (1) too few resources, (2) the need for more personalized teaching approaches, and (3) the tension between cohesion and flexibility. In a Concentration model, faculty serve both a core discipline and global health interests. In terms of resources, alumni were frustrated about the lack of funding in our program for overseas placements and the challenge of few dedicated faculty to provide mentorship or help find placements.

“It’s just frustrating that it’s a global health program, and they do want people to be going abroad, but it’s really difficult financially to do that.”

“There are too few faculty who are engaged with public health in a meaningful way to provide engagement and interaction that would create a dynamic global health environment.”

“As a student, one of the things that I longed for was more mentoring, or some kind of mentoring relationship. And I just don’t think that there’s enough depth or involvement given the number of students or the number of faculty who are actually engaged. It creates a situation where, unfortunately, it’s quite difficult to engage a higher level of learning around global health issues.”

In addition, faculty who serve two masters (discipline and global health) connect and collaborate with people doing global health work within their own disciplines at University of Toronto and across Canada. Alumni observed that:

“There is a real fragmentation of global health work and the global health community at University of Toronto. It’s disturbing to me how many global health centers there are; I mean I think that there may be seven or eight, many of them not communicating with each other, and many of them not connected to the school of public health…There isn’t a sense of cross-pollination and cohesion.”

In a Concentration model, the bulk of the coursework is dedicated to disciplinary skills training so the issue is whether to provide breadth of coverage in the global health courses or depth. Some alumni wanted breadth, others wanted more depth and others wanted both. Some alumni want smaller classes and deeper discussions. Such learning from other students requires them to have experience in the first place and would be more suited to students with medium to higher prior experience.

“As an intro course, I’d probably go more breadth than depth and let people go into more depth with their choice of electives.”

“Most of my learning came from people in the class as opposed to the course material.”

Alumni also expressed a desire for more practical hands-on skills training in global health which is challenging when base program requirements also have to be met.

“Part of what’s been really challenging in terms of finding actual long term employment is that I still don’t have quite enough work experience.”

The current lack of flexibility in the University of Toronto model (daytime program with minimal part-time options) was seen as a drawback by alumni. The demands of the base programs are high and the opportunities to connect to the global health cohort are limited to one core course.

“One of the huge challenges in terms of operationalizing the MPH program is that it’s quite inflexible in terms of its structure. All of the course offerings are during the day, and for me that means not working. I was prepared to do that if the quality of instruction was high, but the equation changes when it is not.”

“Structure the program to take into account professionally activities and things that are currently going on in the world.”

“The people from five different programs will obviously understand the issues differently. There’s a case for interdisciplinary learning, but the structure of the course doesn’t really allow that, so I don’t think that you benefit from having that.”

Some of these comments are related to the start-up phase of a new program (few resources, lack of flexibility), some are related to the differing needs of students with a range of disciplinary interests and prior experiences (teaching approaches), and some are related to the nature of the global health concentration design as a collaborative program (lack of cohesion, few resources).

## 5. Discussion

Because global health is inherently interdisciplinary and multidisciplinary, students from a growing range of disciplines and with a range of levels of experience directly and indirectly related to health seek training (*Crump & Sugarman, 2010)*. The Masters level GH Concentration model at University of Toronto has been able to include students in a diverse range of disciplines such as epidemiology, health promotion, community nutrition, occupational/industrial hygiene, and family and community medicine and accommodate students with a range of prior experience in global health.

On the positive side, at University of Toronto, the GH concentration model builds on strong disciplinary skills in public health and develops broad GH competencies. The review of program monitoring files showed that it was more challenging to meet the GH concentration requirements if the student was studying epidemiology or occupational and environmental health. Most students in the GH concentration admitted from 2006-2009 were classified as having medium to very high levels of prior experience in global health both academically and in terms of work or volunteer experience overseas. There was no clear pattern as to whether those with higher prior experience took more or fewer classes or did more globally-related practica. However, the alumni follow-up data highlighted the role of student prior experience in their subsequent satisfaction with the concentration. Very high prior experience students perhaps had enough global experience and were looking to build discipline-specific skills instead. However, this trend was not significant in the review of program monitoring files. Those dropping the concentration ranged widely in terms of prior experience.

Given our experience of using a concentration model and comments from the alumni survey, the challenges facing a GH concentration model of graduate education are around few resources, need for personalized teaching approaches, flexibility and cohesion. In terms of resources, it is important to recognize the split loyalties of the faculty and the double focus for funding resources which it entails. Trying to provide cohesion across the primary disciplinary programs for students with global health interests is a challenge. In a concentration model, students also have divided loyalties. Alumni did not report strong connections with their global health colleagues. The required course in global public health and optional monthly seminars did not provide enough contact time. Extra efforts need to be made to build a sense of community among GH concentration students.

In considering the challenge of providing personalized teaching approaches and the dilemma of breadth versus depth, we note that a variety of global health electives were available. Students were limited to one to five electives depending on their core program. For students with low prior training or experience, the concentration model may not be able to foster adequate learning in both their base program and global health. For students with very high prior experience, the global health concentration model may not provide enough advanced level learning opportunities. For students with medium to high prior experience, alumni suggested that a better balance of more substantive global electives and small group discussions could deepen learning and enhance their experience.

## 6. Conclusion

The challenge in graduate education is to bring forward the skills and attitudes required for global health work while recognizing that it is not a separate discipline. People with a range of skills are required to work in global organizations or settings. As it currently stands, as an emerging field, global health is an added dimension for all disciplines. This is a central reason why the Dalla Lana School of Public Health chose the concentration model of education. The results reported in this paper lead us to conclude that a concentration model of graduate education is not necessarily better suited to a particular kind of graduate student based on prior experience in global health. However, a concentration model may be able to meet the needs of a range of students, with certain adjustments to the curriculum.

For well connected, experienced students, *mentoring and advanced thinking* may be needed, as they want to be able to grapple with issues they have experienced first hand with other experienced individuals in an academic setting. This group may be satisfied with a *support* model or a *concentration* model where they can focus on learning the primary disciplinary skills they need to apply their understandings of GH. However, they will need advanced courses and opportunities to connect with others with the same level of experience regarding global health.

For students with medium prior experience and training in global health, a *concentration* model with some limited funding may be a good fit. The availability of masters level GH courses and discussions with others having similar experience, drawing on both prior and new links for international practicum placements, should be able to build their competencies. It would enhance the experience for these students to incorporate global health concepts into some of the existing discipline courses in order to increase the attention placed upon GH. Students with low prior experience who want to begin a career in global health, may require more courses and substantial assistance to find international experiences, and hence a GH *dedicated* MPH program could be most suitable.

For a concentration model of graduate education in global health, the full set of global health competencies (Hagopian et al., 2008) could be met for students with medium to high levels of prior experience in global health by providing advanced level courses, small classes with opportunities for deeper discussions, and lots of elective space in the curriculum to meet needs of core discipline and global health. Unless they are registered in a discipline with a lot of electives, those with low prior experience will not be able to meet the basic competencies in a concentration model. Further evaluation research comparing student learning experiences and outcomes across different models of graduate global health education would assist public health educators and prospective students in finding the right match for their global health learning needs.
